# Bioactive Compounds and Antioxidant Activity of Lettuce Grown in Different Mixtures of Monogastric-Based Manure With Lunar and Martian Soils

**DOI:** 10.3389/fnut.2022.890786

**Published:** 2022-04-29

**Authors:** Luigi G. Duri, Antonio Pannico, Spyridon A. Petropoulos, Antonio G. Caporale, Paola Adamo, Giulia Graziani, Alberto Ritieni, Stefania De Pascale, Youssef Rouphael

**Affiliations:** ^1^Department of Agricultural Sciences, University of Naples Federico II, Portici, Italy; ^2^Department of Agriculture, Crop Production and Rural Environment, University of Thessaly, Volos, Greece; ^3^Interdepartmental Research Centre on the “Earth Critical Zone” for Supporting the Landscape and Agroenvironment Management (CRISP), University of Naples Federico II, Portici, Italy; ^4^Department of Pharmacy, University of Naples Federico II, Naples, Italy

**Keywords:** *in situ* resource utilization (ISRU), space farming, mars and lunar simulants, organic amendment, antioxidant activity, carotenoids, phenolic compounds, Orbitrap LC-MS/MS

## Abstract

The supplementation of bioactive compounds in astronaut’s diets is undeniable, especially in the extreme and inhospitable habitat of future space settlements. This study aims to enhance the Martian and Lunar regolith fertility (testing two commercial simulants) through the provision of organic matter (manure) as established by *in situ* resource utilization (ISRU) approach. In this perspective, we obtained 8 different substrates after mixing Mojave Mars Simulant (MMS-1) or Lunar Highlands Simulant (LHS-1), with four different rates of manure (0, 10, 30, and 50%, w/w) from monogastric animals. Then, we assessed how these substrates can modulate fresh yield, organic acid, carotenoid content, antioxidant activity, and phenolic profile of lettuce plants (*Lactuca sativa* L.). Regarding fresh biomass production, MMS-1-amended substrates recorded higher yields than LHS-1-ones; plants grown on a 70:30 MMS-1/manure mixture produced the highest foliar biomass. Moreover, we found an increase in lutein and β-carotene content by + 181 and + 263%, respectively, when applying the highest percentage of manure (50%) compared with pure simulants or less-amended mixtures. The 50:50 MMS-1/manure treatment also contained the highest amounts of individual and total organic acids, especially malate content. The highest antioxidant activity for the ABTS assay was recorded when no manure was added. The highest content of total hydroxycinnamic acids was observed when no manure was added, whereas ferulic acid content (most abundant compound) was the highest in 70:30 simulant/manure treatment, as well as in pure LHS-1 simulant. The flavonoid content was the highest in pure-simulant treatment (for most of the compounds), resulting in the highest total flavonoid and total phenol content. Our findings indicate that the addition of manure at specific rates (30%) may increase the biomass production of lettuce plants cultivated in MMS-1 simulant, while the phytochemical composition is variably affected by manure addition, depending on the stimulant. Therefore, the agronomic practice of manure amendment showed promising results; however, it must be tested with other species or in combination with other factors, such as fertilization rates and biostimulants application, to verify its applicability in space colonies for food production purposes.

## Introduction

In recent years, there is a growing interest in space exploration and the subsequent establishment of extraterrestrial colonies on the Moon or Mars. In addition to the leading space agencies, such as the National Aeronautics and Space Administration (NASA) and the European Space Agency (ESA), private companies (e.g., SpaceX) are also currently focused on space research (1, 2). The synergistic collaboration of many countries and experts in various disciplines has resulted in the achievement of new technological milestones, which should allow the realization of full-fledged missions aimed at both exploration and colonization of new planets in the coming decades (3, 4). When planning the establishment of a future outpost, either on the Moon or Mars, it is fundamental to consider the self-sufficiency of the colony as the hypothesis of total resources that supply from Earth would be practically unrealistic in regard to both time management and high costs (5–7). In this regard, indigenous soil-based agricultural systems could be an effective solution to relieve the inputs of a bioregenerative life support system (BLSS) (8–11), and meanwhile, the *in situ* resource utilization (ISRU) approach could provide a valuable contribution to cost reduction using *in situ* materials and recycling all colony waste products to the maximum extent possible (12–17). Recently, Cannon and Britt (18) evaluated a set of alternatives for the possible development of a self-sufficient community on Mars. The authors assumed the *in situ* production of basic needs, emphasizing that the diet of colony residents would necessarily have to change by focusing on insect farming for food production rather than plant cultivation, pointing out the differences in terms of growth cycle duration, area devoted, and production costs (18). However, this assessment omits the remarkable ecological role that plants may play, since apart from food production, they are able to strongly support the BLSS through water recycling, CO_2_ fixation, and oxygen production (5, 8, 19), thus being a pivotal part of a biological loop that is not only focused on food production. In addition, there are nutritional aspects of plant-derived food products that cannot be neglected, such as their content in fundamental macro- and microminerals, bioactive compounds, such as carotenoids and phenolic acids, that are extremely important for balanced human health (20, 21).

The Martian and Lunar surface is composed mainly of basaltic rocks and sediments that include varying amounts of different minerals, such as olivine, pyroxene, plagioclase, anorthosite, vitric and lithic fragments, iron oxides, and sulfates (22–27). Several studies reported that Martian and Lunar soils are not suitable for plant cultivation, as they are poor in nutrients (primarily nitrogen) and organic matter, and also lack proper soil structure (28, 29). However, these shortcomings could be compensated by proper agronomic practices (30, 31). Due to the unavailability of real Martian and Lunar regolith samples for agronomic testing, scientific experiments on space cultivation can be only conducted with commercial simulants derived from crushed terrestrial rocks, which tend to replicate the geotechnical and compositional characteristics of regolith studied during the past manned and unmanned missions to the Moon or Mars, respectively (17). Over the years, various research organizations have developed different versions of extraterrestrial soil simulants (27, 32). To make such media suitable for plant cultivation, the use of organic soil amendments, such as monogastric manure, could be hypothesized. The use of monogastric manure is based on the concept that this organic matter would be more similar to crew excrement, which after being properly treated (33–35) could be adopted to improve the physicochemical characteristics of regolith. As it is well known, the symbiotic relationship created between plants and the soil microbiomes (e.g., fungi and bacteria) is also fundamental for proper soil fertility. In general, rhizosphere interactions occur through different mechanisms, such as the colonization of root surfaces, bacterial movement, and the creation of synergistic interactions with the plant (36). However, the extreme environment of the Moon and Mars is not conventional for life development; for this reason, the relationship between plant–soil microbiome could be not predictable at planet surface. However, the use of regolith as growing media under “controlled conditions” cannot preclude the establishment of microbial relationships that can improve the fertility of these substrates in a long-term cultivation.

Another important aspect is the species selection for food production in BLSS. The selection of candidate crops was usually carried out using specific criteria, such as nutritional value, plant size, adaptability to extreme environmental conditions, low resource requirements, short crop cycle, and high harvest index (37–41). Among the various candidate species, lettuce (*Lactuca sativa* L.) is highly ranked, as it is a fast-growing leafy vegetable with a high harvest index (> 0.9) (42). In addition, its leaves are rich in mineral elements, dietary fiber, carotenoids, vitamin C, and phenolic compounds (43–47), which are sorely needed especially for space crews subjected to severe oxidative and inflammatory stresses during space missions (48–51).

To date, very few works have evaluated the plant cultivation on Martian or Lunar soil simulants (28, 52, 53). In particular, Gilrain et al. (52) evaluated the Swiss chard (*Beta vulgaris*) growth for the application in Advanced Life Support (ALS) using Martian regolith simulants (JCS-1A Mars) mixed with leaf compost in the following ratios:1:0, 3:1, 1:1, 1:3, and 0:1 (v/v). Onsay et al. (54) assessed the performance of a full crop cycle (of Extra Dwarf pak choi and red romaine lettuce) in Martian regolith simulants (JCS-1A Mars and MGS-1) amended with mushroom compost, measuring biometric parameters and the quantity of chlorophyll and anthocyanin. Finally, Duri et al. (16) grew in a walk-in growth chamber two varieties of lettuce with different leaf pigmentation on four different mixtures (0:100, 70:30, 30:70, and 100:0, v/v) based on Martian simulant (MMS-1) and green compost, and biometric, physiological, and qualitative parameters were measured at harvest. To the best of our knowledge, only these three experiments have adopted regolith–compost mixtures as plant-growing media, providing a Hoagland solution for all experiments, but none of the abovementioned studies addressed the effect of manure amendment. Therefore, the purpose of this work was to evaluate the response in terms of nutraceutical properties of a widely used crop, such as lettuce to soil amendment, by testing mixtures at different percentages of extraterrestrial simulant and monogastric manure. The main aim was to evaluate whether manure amendment can compensate the defects of extraterrestrial regolith, as established in the ISRU approach, to find the best mix that can maximize the nutritional value of lettuce while ensuring an acceptable yield.

## Materials and Methods

### Plant Material, Growth Chamber Condition, and Experimental Treatments

A pot experiment was carried out in an open-gas-exchange growth chamber (28 m^2^: 7.0 m × 2.1 m × 4.0 m; W × H × D; Process-C5, Spagnol srl, Treviso, Italy), with lettuce plants (*Lactuca sativa* L. cv. “Grand Rapids”) grown in different mixtures of extraterrestrial soil simulants and monogastric manure. The lighting of the growth chamber was provided by high-pressure sodium lamps (Master SON-T PIA Plus 400W, Philips, Eindhoven, the Netherlands) with a photosynthetic photon flux density (PPFD) of 420 ± 20 μmolm^–2^ s^–1^. Air temperature was set at 24–18°C (light/dark) with a 12-h photoperiod and a relative air humidity of 60–80% maintained by a fog system. The experiment was carried out at ambient CO_2_ concentration (390 ± 20 ppm), while air exchange and dehumidification were guaranteed by two heating, ventilation, and air conditioning (HVAC) systems. Then, two different simulants were tested: the Mojave Mars Simulant (MMS-1), purchased from The Martian Garden (Austin, Texas, United States) and the Lunar Highlands Simulant (LHS-1) purchased from Exolith Lab (University of Central Florida, Orlando, Florida, United States). Both simulants are coarse-textured alkaline (pH 9 to 10) substrates consisting mostly of plagioclase (anorthite) and amorphous Al and Fe minerals. The tested treatments were prepared after mixing each simulant with sieved (2 mm) horse/swine monogastric manure (Agraria Di Vita srl, Pescia, Pistoia, Italy) at doses of 0, 10, 30, and 50% (w/w). Plants were transplanted into 9 cm x 9 cm x 9 cm pots irrigated with only reverse osmosis water throughout the crop cycle (31 days). The experimental design was laid out according to the randomized complete-block factorial design with four manure amendment rates (M) and two extraterrestrial soil simulants (S), with three replicates. Each experimental plot consisted of four plants (total of 96 plants).

### Sample Preparation, Analysis of Nitrate and Organic Acids

At harvest, the fresh biomass (g plant^–1^) of all plants for each treatment was determined. Fresh samples from each plant were split into two subsamples; one of them was instantly frozen in liquid nitrogen, lyophilized, and stored at –80°C for further phytochemical analyses, whereas the remaining sample was used for water content determination after drying in a forced-air oven at 70°C to constant weight (around 72 h). Oven-dried samples were then ground with a cutting-grinding head mill at 0.5 mm (IKA, MF 10.1, Staufen, Germany) and used for nitrate and organic acid content determinations (malate, tartrate, oxalate, citrate, and isocitrate). In brief, 250 mg of ground leaf tissue was suspended in 50 ml of ultrapure water (Arium^®^ Advance EDI pure water system; Sartorius, Goettingen, Lower Saxony, Germany), stirred in a shaking water bath (ShakeTemp SW22, Julabo, Seelbach, Germany) at 80°C for 10 min, filtered at 0.45 μm, and finally analyzed by ion chromatography (ICS-3000, Dionex, Sunnyvale, CA, United States) as described by Pannico et al. (46).

### Analysis of Antioxidant Activity

For antioxidant activity, 200 mg of lyophilized material was extracted with 5 ml of methanol (stored at 4°C) and then centrifuged at 400 rpm for 5 min. The supernatant was collected and re-centrifuged after adding a further 5 ml methanol to the pellet. A total of two different assays were carried out for antioxidant activity determination: the ABTS-scavenging capacity based on the method described by Re et al. (55), and the 1,1-diphenyl-2-picrylhydrazyl (DPPH) free radical scavenging activity using the procedure proposed by Brand-Williams et al. (56) modified, briefly detailed as follows.

For the ABTS assay, a stock solution was incubated at a temperature of 4°C for 16 h (2.5 ml of aqueous ABTS-7 mM and 44 ml of potassium persulfate-2.45 mM). Then, this stock solution was diluted (1:88) with ethanol having an absorbance of 0.700 ± 0.050 at 734 nm. The analysis was performed by combining 0.1 ml of filtered sample and 1 ml of ABTS radical working solution and then monitoring the absorbance after 2.5 min at 734 nm. For the DPPH assay, 1 ml of methanolic DPPH-100 μm was added to 200 μl of diluted lettuce extract. The absorbance of DPPH was 0.90 ± 0.02 at 517 nm, while the decrease in absorbance of the resulting solution was monitored after 10 min of incubation at room temperature in the dark at 517 nm. All determinations were performed in triplicate, and the results were expressed as Trolox^®^ equivalent antioxidant capacity (TEAC, mmol Trolox^®^ equivalents kg^–1^ dw).

### Extraction and Preparation for Carotenoids and Phenolic Profile Assays

The freeze-dried lettuce samples (100 mg) were extracted by modifying the method of Kim et al. (57). The samples were mixed in 6 ml of ethanol containing 0.1% BHT and placed in a water bath for 5 min at 85°C. Then, 120 μl of 80% KOH was added to the samples, and subsequently, they were vortexed and returned to the water bath for 10 min at the same temperature. In the end, they were placed in ice to stop the reaction and in each solution were added 3 ml of distilled water and 3 ml of hexane. Subsequently, centrifugation was applied to collect the hexane layer, and the pellet was re-extracted two times more using hexane. Finally, after all the extraction procedures, the hexane layers were combined and dried with nitrogen gas. About 1 ml of chloroform was added to recover the residue and filtered with a 0.2-μm nylon filter before the quantification by HPLC-DAD. For the quantification, a Shimadzu HPLC Model LC 10 (Shimadzu, Osaka, Japan) was used, equipped with a reverse-phase 250 mm × 4.6 mm, 5 μm Gemini C18 column (Phenomenex, Torrance, CA, United States), after injecting 20 μl of sample. The following A:B gradient: 0–8 min (82:18); 8–12 min (76:24); 12–18 min (39:61); and 18–25 min, a linear gradient to equilibration (82:18), was prepared using acetonitrile and ethanol/n-hexane/dichloromethane (1:1:1) (respectively, for mobile phases A and B). The absorbance measurements were recorded at 450 nm and expressed in mg kg^–1^ dw. To perform the quantification, a linear calibration curve was carried out using lutein and β-carotene standards at 6 levels of concentration (from 5 up to 100 μg ml^–1^). About 100 mg of lyophilized sample was used for polyphenol quantification (expressing it as μg g^–1^ dw). The extraction procedure involved the use of 5 ml of methanol and water solution (60:40, v/v), which was sonicated with the sample for 30 min. Then, the suspension was centrifuged (400 rpm) for 15 min and filtered with filter paper (0.45 μm) using 10 μl for mass spectrometry (HRMS-Orbitrap) analysis. UHPLC system (UHPLC, Thermo Fisher Scientific, Waltham, MA, United States) equipped with a Dionex Ultimate 3000 Quaternary pump performing at 1,250 bar and a thermostated (25°C) Kinetex 1.7 μm biphenyl (100 mm × 2.1 mm) column (Phenomenex, Torrance, CA, United States) was used to carry out the polyphenol determination assay. A volume of 2 μl was injected, using a flow rate of 0.2 ml min^–1^ to elute and using 0.1% formic acid in water and 0.1% formic acid in methanol, respectively, to prepare a gradient of A and B in the following way: 0 min – 5% B, 1.3 min – 30% B, 9.3 min – 100% B, 11.3 min – 100% B, 13.3 min – 5% B, and 20 min – 5% B. A Q Exactive Orbitrap LC-MS/MS (Thermo Fisher Scientific, Waltham, MA, United States) was involved in mass spectrometry analysis. An ESI source (HESI II, Thermo Fischer Scientific, Waltham, MA, United States) in negative ion mode (ESI-) was used. The following ion source parameters were applied: −2.8 kV spray voltage, sheath gas (N_2_ > 95%) 45, auxiliary gas (N_2_ > 95%) 10, capillary temperature 275°C, S-lens RF level 50, and auxiliary gas heater temperature 305°C. The polyphenolic compound targeted acquisition was carried out on parallel reaction monitoring (PRM) mode, set as follows: microscans at 1, resolution at 35.000, AGC target at 5e5, maximum ion time at 100 ms, MSX count at 1, and isolation window at 1.0 m/z. The input time frame for elution and collision energy (CE) were optimized for each polyphenolic compound. The accuracy and calibration of the Q Exactive Orbitrap LC-MS/MS were checked using a Thermo Fisher Scientific reference standard mixture and setting the mass tolerance window for the two analysis modes at 5 ppm. The linearity of the method for both low and high (5 mg kg^–1^–120 mg kg^–1^) concentration ranges was assessed using six concentration levels in each calibration range. The low limit of detection (LOD) and low limit of quantitation (LOQ) values for HPLC-DAD analysis of carotenoids were determined for β-carotene, while in the case of LC-MS/MS analysis of polyphenols were based on chlorogenic acid and rutin signal-to-noise levels. LOD and LOQ for each compound were obtained by serial dilutions of stock solution, and the analysis and processing of data were performed using the Xcalibur software, v. 3.0.63 (Thermo Fisher Scientific, Waltham, MA, United States).

### Statistical Analysis

Data were subjected to two-way ANOVA using the IBM SPSS software package (SPSS Inc., Chicago, Illinois, United States). The mean effects of simulants (S) and manure amendment (M) factors were compared according to the unpaired Student’s *t*-test and one-way analysis of variance, respectively. Significant statistical differences were determined by Tukey–Kramer HSD test for the S factor and the S × M interaction at the level of *p* ≤ 0.05.

## Results

### Fresh Biomass, Nitrate, and Organic Acid Contents

**FIGURE 1:**
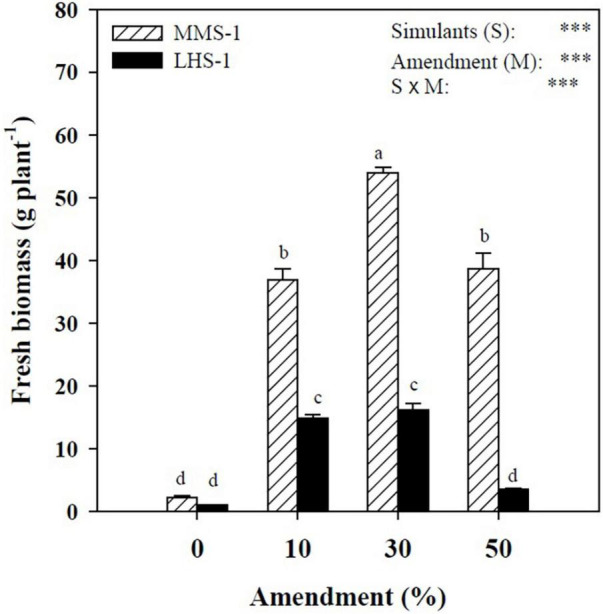
Fresh biomass of lettuce grown in different mixtures of MMS-1 or LHS-1 simulants and manure (simulant/manure rates: 100:0, 90:10, 70:30, 50:50; w/w).

A statistically significant interaction was observed between simulant (S) and manure percentage (M) factors, with the highest value recorded in the treatment where MMS-1 was combined with 30% manure, while the Lunar simulant (LHS-1) had the highest yields in the intermediate treatments (10 and 30% of manure) (Figure 1). Moreover, regardless of the amendment treatment, lettuce fresh biomass produced on the MMS-1 simulant was on average of 3.7-folds higher than on LHS-1, indicating that the specific simulant is more appropriate for lettuce cultivation than the LHS-1 substrate.

The simulant mean effect showed significant differences for the content of water, nitrate, malate, citrate, and isocitrate, with the highest values obtained in MMS-1 except for nitrate content which was significantly lower compared to lunar simulant (−22%) (Table 1). The water content showed a direct correlation with the percentage of amendment (*R* = 0.89) presenting a 24% increase in the 50% manure treatments compared to the non-amended simulants (Table 1).

**TABLE 1:** Water, nitrate, and organic acid contents of grown lettuce in different mixtures of MMS-1 or LHS-1 simulants and manure (simulant/manure rates: 100:0, 90:10, 70:30, 50:50; w/w).

Source of variance		Water content	Nitrate	Malate	Tartrate	Oxalate	Citrate	Isocitrate
		(%)	mg kg^–1^ fw	g kg^–1^ dw				
Simulants (S)	MMS-1	84.1 ± 1.83 a	12.49 ± 0.58 b	25.36 ± 4.13 a	3.15 ± 0.38	0.77 ± 0.05	12.64 ± 0.28 a	0.268 ± 0.012 a
	LHS-1	81.9 ± 2.21 b	15.91 ± 1.02 a	20.26 ± 2.71 b	2.80 ± 0.27	0.76 ± 0.02	11.81 ± 0.35 b	0.234 ± 0.012 b
Amendment (%) (M)	0	72.3 ± 1.13 d	14.59 ± 1.19	7.05 ± 0.33 d	1.48 ± 0.09 c	0.73 ± 0.04 bc	12.05 ± 0.64	0.265 ± 0.006 ab
	10	83.1 ± 0.63 c	13.19 ± 1.45	20.63 ± 0.86 c	2.95 ± 0.21 b	0.68 ± 0.03 c	12.12 ± 0.39	0.241 ± 0.029 bc
	30	86.8 ± 0.62 b	13.33 ± 1.15	27.96 ± 1.47 b	3.37 ± 0.11 b	0.79 ± 0.02 ab	12.68 ± 0.48	0.220 ± 0.008 c
	50	89.7 ± 0.73 a	15.68 ± 1.66	35.59 ± 4.58 a	4.12 ± 0.41 a	0.85 ± 0.08 a	12.06 ± 0.42	0.278 ± 0.010 a
S × M	MMS-1 × 0	74.7 ± 0.17	12.74 ± 1.83	7.48 ± 0.44 c	1.54 ± 0.18 c	0.65 ± 0.01 cd	13.30 ± 0.63 ab	0.259 ± 0.009 abc
	MMS-1 × 10	83.5 ± 0.32	11.75 ± 0.86	20.71 ± 1.35 b	3.04 ± 0.44 b	0.61 ± 0.01 d	12.46 ± 0.62 ab	0.303 ± 0.014 a
	MMS-1 × 30	87.2 ± 0.76	12.62 ± 0.90	29.37 ± 2.87 b	3.15 ± 0.06 b	0.78 ± 0.04 bc	11.98 ± 0.57 ab	0.214 ± 0.009 cd
	MMS-1 × 50	90.9 ± 0.94	12.85 ± 1.50	43.86 ± 3.98 a	4.89 ± 0.33 a	1.02 ± 0.06 a	12.83 ± 0.36 ab	0.297 ± 0.006 a
	LHS-1 × 0	69.9 ± 0.80	16.44 ± 0.57	6.62 ± 0.39 c	1.42 ± 0.04 c	0.80 ± 0.02 b	10.80 ± 0.35 b	0.272 ± 0.005 ab
	LHS-1 × 10	82.8 ± 1.33	14.64 ± 2.78	20.55 ± 1.35 b	2.86 ± 0.17 b	0.75 ± 0.02 bcd	11.79 ± 0.51 ab	0.179 ± 0.010 d
	LHS-1 × 30	86.4 ± 1.09	14.05 ± 2.29	26.55 ± 0.81 b	3.59 ± 0.12 b	0.81 ± 0.02 b	13.39 ± 0.57 a	0.225 ± 0.015 bcd
	LHS-1 × 50	88.4 ± 0.48	18.52 ± 1.88	27.31 ± 4.54 b	3.36 ± 0.35 b	0.68 ± 0.03 bcd	11.29 ± 0.37 ab	0.258 ± 0.009 abc
Significance	S	**	*	**	ns	ns	*	***
	M	***	ns	***	***	***	ns	***
	SxM	ns	ns	*	**	***	**	***

ns,*,**, *** Non-significant or significant at p ≤ 0.05, 0.01, and 0.001, respectively. Cultivar means were compared by t-test. Substrate mixture means and interaction were compared by Tukey’s multiple-range test (p = 0.05). Different letters within each column indicate significant differences.

A statistically significant interaction of the two factors (S × M) was observed in the content of all detected organic acids. There was a significantly higher content of malate, tartrate, and oxalate in the 50% MMS-1 mixture compared to the rest of the treatments where an increase by 486, 217, and 67% was recorded, respectively; however, the citrate content did not differ between the various combinations of simulants and manure rates (Table 1). Regarding the Lunar simulant, malate and tartrate contents were significantly higher (on average of + 275 and + 130%, respectively) in the amended mixtures than in the pure LHS-1. On the other hand, the isocitrate content was significantly higher in the non-amended simulant and 50% amendment than in the 10 and 30% manure treatment; in contrast, the citrate content was significantly lower in the non-amended LHS-1 compared to the 30% amended mixture (Table 1). Finally, the highest content of individual and total organic acids (except for the case of citrate where no significant differences were observed) was recorded for the highest rate (50%) of manure, regardless of the simulant, whereas MMS-1 had higher amounts of malate, citrate, and isocitrate than the LSH-1 simulant, regardless of the manure rate (Table 1).

### Antioxidant Activity

A significant interaction of the two factors (S × M) was observed for ABTS and DPPH assays, where the non-amended simulants had the highest Trolox content in the case of ABTS, whereas the non-amended simulants and the combination of MMS-1 × 50% of manure and LSH-1 × 30% of manure recorded the highest activity in DPPH assay (Table 2). The simulant mean effect (S) showed significantly higher ABTS and DPPH assays in lettuce grown on LHS-1 (+ 3.2 and + 10.7%, respectively) compared with those on MMS-1 (Table 2). Similarly, the non-amended simulants showed the highest activity in all the studied assays.

**TABLE 2:** Antioxidant activity of lettuce grown in different mixtures of MMS-1 or LHS-1 simulants and manure (simulant/manure rates: 100:0, 90:10, 70:30, 50:50; w/w).

Source of variance		ABTS	DPPH
		mmol Trolox kg^–1^	
Simulants (S)	MMS-1	74.97 ± 3.9 b	56.17 ± 2.1 b
	LHS-1	77.38 ± 4.1 a	62.17 ± 1.3 a
Amendment (%) (M)	0	97.95 ± 1.3 a	66.40 ± 1.6 a
	10	66.46 ± 3.0 c	54.20 ± 2.6 c
	30	68.40 ± 0.7 bc	57.03 ± 2.9 bc
	50	71.90 ± 1.8 b	59.04 ± 0.6 b
S × M	MMS-1 × 0	95.44 ± 1.1 a	64.81 ± 1.9 ab
	MMS-1 × 10	60.80 ± 1.6 d	48.90 ± 1.8 d
	MMS-1 × 30	67.80 ± 1.0 cd	50.89 ± 2.3 cd
	MMS-1 × 50	75.86 ± 0.7 b	60.07 ± 0.4 ab
	LHS-1 × 0	100.4 ± 0.9 a	67.99 ± 2.6 a
	LHS-1 × 10	72.13 ± 3.0 bc	59.50 ± 1.3 b
	LHS-1 × 30	69.00 ± 1.1 bc	63.16 ± 0.5 ab
	LHS-1 × 50	67.95 ± 0.6 c	58.02 ± 0.6 bc
Significance	S	*	***
	M	***	***
	SxM	***	**

*,**, *** Significant at p ≤ 0.05, 0.01, and 0.001, respectively. Cultivar means were compared by t-test. Substrate mixture means and interaction were compared by Tukey’s multiple-range test (p = 0.05). Different letters within each column indicate significant differences.

### Carotenoid Content

**FIGURE 2:**
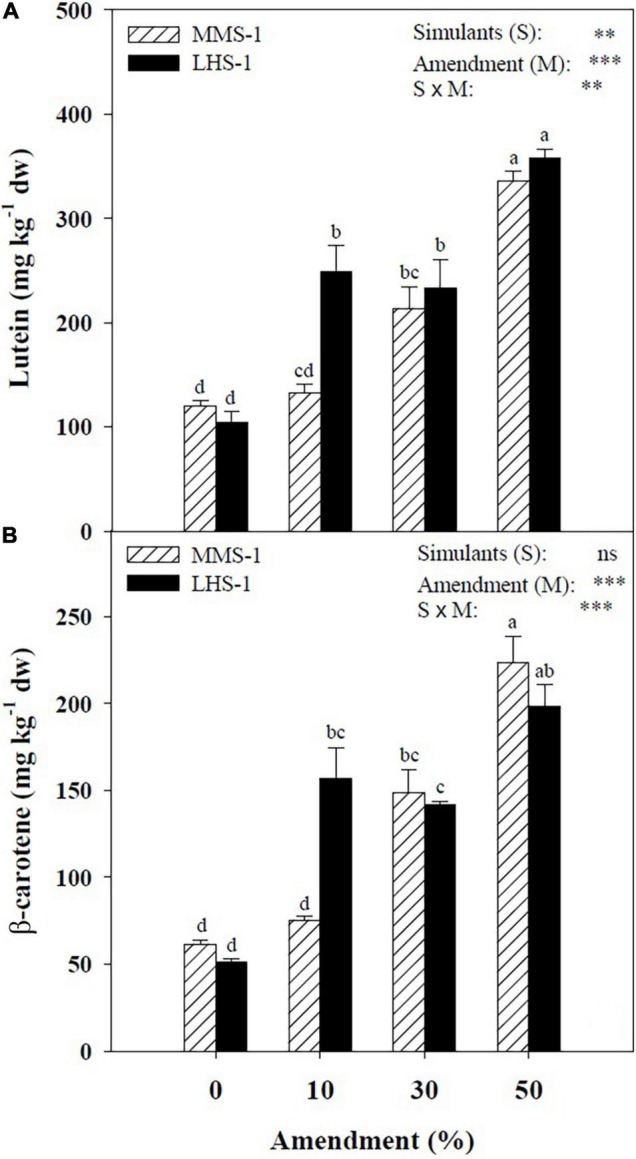
Lutein and β-carotene concentration of lettuce grown in different mixtures of MMS-1 or LHS-1 simulants and manure (simulant/manure rates: 100:0, 90:10, 70:30, 50:50; w/w).

The mean effect of simulants (S) showed a significantly higher concentration of lutein in LHS-1 (236 mg kg^–1^) compared to MMS-1 (200 mg kg^–1^), whereas no significant difference was detected in β-carotene content. For both carotenoids, a direct correlation was observed between their content and the amendment percentage of the different mixtures (*R* > 0.97). Moreover, a significant interaction of S × M was found in both carotenoids reaching the highest content at the 50% manure dose (Figure 2). Specifically, the Martian simulant recorded an increase in lutein and β-carotene content at 30 (+ 78.7 and + 141%, respectively) and 50% (+ 181 and + 263%, respectively) amendment compared to non-amended MMS-1 (Figure 2). Regarding LHS-1, the lutein content is on average of 132% higher at the two intermediate mixtures and 245% higher at the maximum manure dose with respect to the non-amended simulant. Similarly, the β-carotene content in the lunar simulant was significantly higher by 206 and 287% at 10 and 50% amendment treatments, respectively, whereas its concentration at the 30% manure dose was assessed between the latter two mixtures (Figure 2).

### Phenolic Compound Profile and Total Phenolic Composition

Chlorogenic acid was the most prevalent compound among all detected hydroxycinnamic acids, followed by ferulic acid, feruloyl-disinapoyl-gentiobiose, and synapoyl-hexose (Table 3A). A significant interaction of S × M factors was found for coumaroyl-diglucoside, ferulic acid, feruloyl-disinapolyl-gentiobiose, and synapoyl-hexose. Specifically, coumaroyl-diglucoside was the highest in the non-amended MMS-1 simulant, whereas the content of feruloyl-disinapolyl-gentiobiose results higher in both pure simulants. Instead, ferulic acid and feruloyl-disinapoyl-gentiobiose varied among the treatments. Ferulic acid was significantly higher in the pure LHS-1 compared to the 10 and 50% manure mixtures. In contrast, synapoyl-hexose was significantly higher in the 30 and 50% manure Martian simulant than in pure MMS-1. The mean effect of simulants (S) was significant for coumaroyl-diglucoside and synapoyl-hexose content which was the highest in MMS-1, whereas the opposite trend was found for ferulic acid (highest content in LSH-1 simulant). The mean effect of the amendment (M) revealed an inverse correlation between chlorogenic acid concentration and manure dose in the different mixtures, reaching a 20.7% reduction at the highest amendment percentage compared to the non-amended simulant. The same trend was also observed for total hydroxycinnamic acids, whereas the opposite trend was recorded for synapoyl-hexose which increased in the amended simulant compared to the non-amended one (Table 3A).

**TABLE 3A:** Phenolic profiles and total phenolic composition of lettuce grown in different mixtures of MMS-1 or LHS-1 simulants and manure (simulant/manure rates: 100:0, 90:10, 70:30, 50:50; w/w).

Source of variance		Chlorogenic acid	Coumaroyl-diglucoside	Ferulic acid	Feruloyl-disinapoyl-gentiobiose	Synapoyl-hexose	Total hydroxycinnamic acids
		μ g g^–1^ dw					
Simulants (S)	MMS-1	2545 ± 67	0.155 ± 0.034 a	45.41 ± 2.59 b	6.77 ± 0.33	8.16 ± 0.42 a	2605 ± 66.8
	LHS-1	2639 ± 87	0.083 ± 0.012 b	55.67 ± 4.26 a	6.85 ± 0.49	6.94 ± 0.38 b	2708 ± 90.2
Amendment (%) (M)	0	2951 ± 81 a	0.247 ± 0.045 a	59.57 ± 8.18 a	8.92 ± 0.30 a	6.54 ± 0.29 b	3027 ± 89.0 a
	10	2583 ± 47 b	0.067 ± 0.003 b	41.31 ± 1.89 c	5.67 ± 0.25 c	8.21 ± 0.29 a	2638 ± 46.0 b
	30	2492 ± 56 bc	0.078 ± 0.015 b	55.86 ± 3.24 ab	6.77 ± 0.10 b	8.42 ± 0.68 a	2563 ± 53.0 bc
	50	2341 ± 48 c	0.084 ± 0.012 b	45.40 ± 2.28 bc	5.89 ± 0.21 c	7.04 ± 0.78 ab	2400 ± 47.7 c
S × M	MMS-1 × 0	2817 ± 102	0.343 ± 0.026 a	43.60 ± 4.97 b	8.34 ± 0.33 a	6.04 ± 0.40 bc	2875 ± 106
	MMS-1 × 10	2542 ± 94	0.061 ± 0.001 cd	41.35 ± 3.61 b	5.52 ± 0.42 cd	8.56 ± 0.15 ab	2598 ± 91.1
	MMS-1 × 30	2528 ± 70	0.106 ± 0.016 bc	55.09 ± 5.89 ab	6.89 ± 0.16 b	9.33 ± 0.76 a	2600 ± 64.8
	MMS-1 × 50	2292 ± 73	0.111 ± 0.001 bc	41.59 ± 3.31 b	6.35 ± 0.10 bcd	8.72 ± 0.22 a	2348 ± 70.1
	LHS-1 × 0	3086 ± 68	0.151 ± 0.007 b	75.54 ± 7.43 a	9.50 ± 0.13 a	7.03 ± 0.08 abc	3178 ± 73.7
	LHS-1 × 10	2623 ± 28	0.073 ± 0.002 cd	41.28 ± 2.21 b	5.82 ± 0.33 bcd	7.86 ± 0.53 abc	2678 ± 26.3
	LHS-1 × 30	2455 ± 96	0.049 ± 0.000 d	56.63 ± 4.15 ab	6.65 ± 0.09 bc	7.51 ± 0.95 abc	2526 ± 92.0
	LHS-1 × 50	2391 ± 61	0.058 ± 0.006 cd	49.22 ± 0.65 b	5.44 ± 0.11 d	5.36 ± 0.38 c	2451 ± 61.9
Significance	S	ns	***	**	ns	**	ns
	M	***	***	**	***	**	***
	SxM	ns	***	**	**	**	ns

ns, **, *** Non-significant or significant at p ≤ 0.01 and 0.001, respectively. Cultivar means were compared by t-test. Substrate mixture means and interaction were compared by Tukey’s multiple-range test (p = 0.05). Different letters within each column indicate significant differences.

Regarding the flavonoid profile, no significant interactions S x M were found in flavonoids as well as the content of total flavonoids and total phenols. Moreover, the mean effect of simulants (S) showed a significantly higher concentration in the case of kaempferol-3-diglucoside, quercetin 3-sophoroside-7-glucoside, and rutin in LHS-1 compared to MMS-1 simulant (Table 3B). Interestingly, the mean effect of the amendment (M) revealed a progressive decrease in the content of most of the detected compounds when manure was added in the tested simulants. The same trend was also observed for the content of total flavonoids and total phenols, resulting in a reduction at the maximum manure dose of 53.0 and 23.3%, respectively, compared to the non-amended simulant (Table 3B).

**TABLE 3B:** Phenolic profiles and total phenolic composition of lettuce grown in different mixtures of MMS-1 or LHS-1 simulants and manure (simulant/manure rates: 100:0, 90:10, 70:30, 50:50; w/w).

Source of variance		Hyperoside	Km 3-diglucoside	Kaempferol-3-glucoside	Quercetin -3-glucoside	Qn 3-sophoroside- 7-glucoside	Rutin	Luteolin-7- O-glucoside	Total flavonoids	Total phenols
		μ g g^–1^ dw								
Simulants (S)	MMS-1	158.8 ± 16.4	3.43 ± 0.47 b	1.45 ± 0.06	17.21 ± 1.67	0.690 ± 0.143 b	3.24 ± 0.44 b	1.63 ± 0.07	186.4 ± 19	2792 ± 84.5
	LHS-1	168.5 ± 11.1	4.06 ± 0.55 a	1.36 ± 0.09	18.03 ± 1.20	0.854 ± 0.141 a	3.89 ± 0.52 a	1.60 ± 0.14	198.3 ± 13	2907 ± 103
Amendment (%) (M)	0	221.5 ± 5.7 a	6.54 ± 0.38 a	1.29 ± 0.13	23.83 ± 0.58 a	1.503 ± 0.102 a	6.20 ± 0.37 a	1.41 ± 0.14	262.3 ± 6.1 a	3289 ± 92 a
	10	165.9 ± 11.4 b	3.00 ± 0.15 b	1.46 ± 0.03	17.83 ± 1.11 b	0.720 ± 0.057 b	2.82 ± 0.14 b	1.67 ± 0.04	193.4 ± 13 b	2831 ± 58 b
	30	163.8 ± 6.9 b	3.03 ± 0.21 b	1.33 ± 0.09	17.37 ± 0.80 b	0.548 ± 0.095 bc	2.86 ± 0.19 b	1.52 ± 0.11	190.5 ± 7.9 b	2753 ± 58 bc
	50	103.4 ± 11.7 c	2.41 ± 0.21 b	1.56 ± 0.12	11.45 ± 1.05 c	0.315 ± 0.019 c	2.37 ± 0.23 b	1.84 ± 0.25	123.3 ± 13 c	2523 ± 58 c
S × M	MMS-1 × 0	220.9 ± 10.7	5.97 ± 0.46	1.52 ± 0.13	23.75 ± 1.15	1.473 ± 0.099	5.63 ± 0.44	1.64 ± 0.15	260.9 ± 12	3136 ± 118
	MMS-1 × 10	163.5 ± 24.8	2.87 ± 0.24	1.50 ± 0.05	17.65 ± 2.44	0.622 ± 0.022	2.69 ± 0.18	1.63 ± 0.06	190.4 ± 28	2788 ± 119
	MMS-1 × 30	168.3 ± 14.5	2.82 ± 0.20	1.24 ± 0.12	17.77 ± 1.72	0.339 ± 0.026	2.66 ± 0.18	1.50 ± 0.15	194.6 ± 17	2794 ± 78
	MMS-1 × 50	82.47 ± 7.8	2.06 ± 0.13	1.56 ± 0.10	9.66 ± 0.88	0.325 ± 0.002	1.97 ± 0.11	1.75 ± 0.18	99.80 ± 9.0	2448 ± 70
	LHS-1 × 0	222.1 ± 6.9	7.12 ± 0.45	1.06 ± 0.13	23.91 ± 0.62	1.534 ± 0.202	6.76 ± 0.42	1.19 ± 0.13	263.6 ± 6.8	3442 ± 72
	LHS-1 × 10	168.4 ± 5.0	3.12 ± 0.20	1.43 ± 0.01	18.01 ± 0.47	0.819 ± 0.078	2.95 ± 0.23	1.71 ± 0.04	196.4 ± 5.4	2874 ± 28
	LHS-1 × 30	159.3 ± 2.1	3.23 ± 0.37	1.42 ± 0.13	16.97 ± 0.27	0.756 ± 0.022	3.06 ± 0.32	1.55 ± 0.19	186.3 ± 3.0	2712 ± 95
	LHS-1 × 50	124.3 ± 13.8	2.76 ± 0.30	1.55 ± 0.24	13.25 ± 1.23	0.306 ± 0.040	2.77 ± 0.30	1.92 ± 0.51	146.9 ± 16	2598 ± 78
Significance	S	ns	*	ns	ns	*	**	ns	ns	ns
	M	***	***	ns	***	***	***	ns	***	***
	SxM	ns	Ns	ns	ns	ns	ns	ns	ns	ns

ns,*,**, *** Non-significant or significant at p ≤ 0.05, 0.01, and 0.001, respectively. Cultivar means were compared by t-test. Substrate mixture means and interaction were compared by Tukey’s multiple-range test (p = 0.05). Different letters within each column indicate significant differences.

## Discussion

This study evaluates the possibility of using human excreta (replaced by a surrogate derived from monogastric farm animals) as a soil amendment to improve the physicochemical and structural characteristics of Martian or Lunar regolith for plant production, in the preparation for a future manned mission to Mars or Moon, respectively. To improve ISRU protocols and minimize procurement from the Earth, the impact of the amendment rate on the nutritional and functional characteristics of lettuce was evaluated. Although this vegetable provides only a limited amount of calories, it is nevertheless a rich source of bioactive compounds, such as carotenoids (mainly lutein and β-carotene) and polyphenols, which counteract the development of chronic diseases and could be helpful to maintain health in space missions (58). A long-term space mission inevitably results in a progressive decline in astronauts’ mental and physical performances (59). In this context, dietary supplementation with fresh vegetables rich in nutraceuticals possessing high antioxidant activity could, on the one hand, minimize pathophysiological effects (59, 60) and, on the other hand, help maintain mental well-being of individuals forced to live in isolated or extreme environments (17, 50). Considering the above, two commercial simulants (a Martian and a Lunar simulant) were amended with different ratios of manure from monogastric animals aiming to evaluate the possible effects on lettuce cultivation. Although these simulants can be a source of available nutrients, such as Ca, Mg, and K, they lack organic C, N, and available P and S, essential for plant growth. As well, they easily release soluble Na, which can induce salt stress in the plants [(61), submitted]. Whereas the manure was characterized by a low C/N atomic ratio (i.e., 11.0 ± 0.4), it can provide a significant amount of potentially available N for rhizosphere microorganisms and plant roots. At the same time, according to the mediumlow H/C atomic ratio (i.e., 1.5 ± 0.1), it would comprise a significant aromatic moiety, ensuring good stability of the organic matter over time. It is also an important source of nutrients; however, it contains significant amounts of Na, which negatively raise its pH to 9.0 and E.C. to ∼7 dS m^–1^ [(61), submitted]. According to the literature, the previous experiment with human excreta as plant fertilizers gave promising results in terms of crop performance and acceptability from farmers (33). Therefore, the goal of this experiment was to test whether it is possible to improve the physicochemical parameters of Martian and Lunar simulants through the manure amendment and extrapolate the obtained results to the cultivation of lettuce in space conditions using human excreta as a soil amendment.

Regardless of treatments, the low yield recorded was attributable to a severe nutritional deficiency as excluding the limited input by the manure, both regolith simulants were highly lacking in key macronutrients and organic matter (61, 62). The higher fresh biomass obtained in plants grown on the Martian simulant could be due to the worse physicochemical characteristics of the Lunar substrate, such as the lower water retention capacity and higher pH, and/or the higher ammonium nitrate content of the Martian regolith, as assessed in a complementary study – part 1 [(61), submitted] and discussed as well by Wamelink et al. (28). By analyzing the interaction between the tested factors (S x M), the yield reduction observed in plants grown on the substrates containing 50% of manure, especially in the case of LHS-1 simulant, could be due to the increase in electrical conductivity of the substrates by the higher manure content (63). This trend was also analyzed and discussed in a complementary study – part 2 [(64), submitted], which assessed the suitability of these eight MMS-1 or LHS-1/manure mixtures for space food production, by matching their physicochemical and hydraulic characteristics with the lettuce growth performance (biometric and physiological parameters), soil enzymatic activity, and nutrient bioavailability in the growth media at plant harvest time.

According to Wamelink et al. (28), the better performance of the Martian simulant compared to the Lunar one could be due to better water-holding capacity; however, the increased rates of manure (> 30%) probably exaggerated the water content and had negative effects on lettuce plants grown in both simulants. Similar results were also observed by Petropoulos et al. (65) who also associated the differences in water content of lettuce plants to the differences in water-holding capacity of the substrates tested. The results of our study are in accordance with the findings of Duri et al. (16) who also suggested that the amendment of Martian regolith with 30% of compost was the most realistic in terms of crop performance and compost availability in space conditions, whereas the 30:70 (Martian regolith/compost) gave the best overall results. Caporale et al. (29) also suggested the use of 30% of green compost due to larger leaf area and better pore size distribution. However, Duri et al. (16) also suggested that a genotype-dependent response was observed, which could justify the differences with our study. Moreover, the abovementioned studies used composts as soil amendments instead of manure, and this could also explain the differences in observed results due to the difference in the physicochemical properties of the tested amendments. Therefore, it seems that the intermediate amounts of manure are the most beneficial since they not only increase the amounts of water that simulants may hold but also increase nutrient availability and retain pH and EC values at acceptable levels for efficient plant growth.

The percentage of manure amendment in the substrates also affected the plant antioxidant activity, showing a clear response to nutritional stress, confirming the findings observed in previous work (16). The non-amended simulants showed the highest antioxidant activity in the case of ABTS assay, whereas the response to DPPH varied among the tested treatments. Moreover, in all the assays, the non-amended simulants had the highest antioxidant activity, regardless of the simulant which further justifies our previous argument regarding the stressful conditions that lettuce plants were subjected to when grown in non-amended substrates. Our hypothesis was also supported by the content of phenolic acid, flavonoid, and total phenol, since the biosynthesis of these secondary metabolites tends to increase as a response to plant stressors (66), while their content is strongly dependent on genotype and agronomic conditions (67, 68). Moreover, the higher ABTS and DPPH values recorded in lettuces grown on LHS-1 mixtures were consistent with the lower fresh biomass yield for the same treatment, confirming the high stress exerted by the lunar simulant.

In agreement with Kim et al. (58), the most abundant phenolic acid in lettuce in our study was chlorogenic acid, which indicates that the phenolic profile is highly associated with the genotype (45, 69). Moreover, the content of total phenolic acids, total flavonoids, and total phenols was the highest in non-amended simulants which was also reported by Duri et al. (16) for red Salanova lettuce plants, whereas the same authors did not record significant differences in total phenol content for green Salanova plants at the tested rates of simulant/compost. This finding indicates that genotype is also important for plant response to abiotic stressors, and various lettuce genotypes should be tested in future studies to make safe conclusions about the potential of cultivating lettuce in Martian or Lunar soils and the possibility to use human excreta as soil amendments.

Nitrate content in leaf tissues is strongly influenced by soil nitrate and ammonium levels (70); nevertheless, in our experiment, the amendment dose did not significantly affect the foliar concentration of this anion. Regarding the effect of the simulant, two hypotheses can explain the lower nitrate content recorded in MMS-1. The first is a probable dilution effect triggered by both the higher fresh biomass and the greater water content of plants grown on this simulant compared to LHS-1; the second hypothesis considers a lower accumulation due to a higher rate of assimilation into organic compounds (such as proteins and nucleic acids). Very low nitrate levels (on a fresh basis) have also been found in lettuce (71) and *Brassicaceae* (72) grown under severe nutrient deficit. However, it seems that nitrate content is also highly depended on the genotype since according to El-Nakhel et al. (37), significant differences between two Salanova lettuce cultivars (green and red Salanova) showed a different response to nitrate accumulation when grown under controlled conditions. It is also important to facilitate optimum growth conditions, especially regarding light intensity, since leafy vegetables tend to increase its content when grown under suboptimal light conditions (11, 73).

Manures have been shown to improve the water retention capacity of substrates (17, 74). These findings corroborate the higher water content of lettuce plants recorded at high manure doses, probably due to increased water availability as the percentage of amendment increases [(54), submitted]. The dose of manure in regolith mixtures also induced an adaptive response by the plants, which resulted in an accumulation of organic acids. These metabolites are involved in different biochemical pathways at the cell level, as the intermediates of photosynthesis and amino acid biosynthesis (75, 76) or as osmoregulators and cell protectors against stress conditions (77). In our experiment, the increased content of malate, tartrate, oxalate, and isocitrate in the 50% substrates is likely due to higher salt stress, as observed in previous work on lettuce (78) and other leafy vegetables (79). Moreover, the content of specific organic acids, such as oxalates, is also associated with nitrogen availability and nitrogen form, and an increase of oxalates should be expected with increasing nitrogen availability (77, 80), as was also the case of high amendment rates in our study. The content of organic acids in vegetables not only affects their taste, but also their acceptability, nutraceutical value, and shelf life (81–83). In addition, they are beneficial to human health by acting as antioxidants due to their ability to chelate metals (84).

The increase in lutein and β-carotene achieved in plants grown on the 50:50 mixtures was consistent with findings on lettuce by Kim et al. (58) following increasing doses of soil amendment. In the same line, Hernández et al. (85) suggested that reduced nitrogen availability resulted in reduced amounts of carotenoids, whereas high salinity induced the biosynthesis of these compounds. High amounts of carotenoids, such as lutein and β-carotene, may play a protective role against oxidative stress since they act as reactive oxygen species scavengers and quenchers of free radicals (11). Although carotenoid content is highly associated with light conditions and light intensity in particular (86, 87), the application of soil amendments from different sources may also increase their content and improve the quality of lettuce plants (88) and their health beneficial effects (58). However, considering the genotypic variation in carotenoid content among the various lettuce genotypes, further studies with multiple genotypes are needed to identify those cultivars that could be used in space colonies as health-promoting food sources (89).

## Conclusion

The supplementation of bioactive compounds in astronaut’s diets is undeniable, especially in the extreme and inhospitable habitat of future space settlements. The carotenoid content was positively correlated with the increment of monogastric manure in the growth substrate (+ 210% of lutein and + 273% of β-carotene). In contrast, the content of total phenols was lower in amended simulants than in pure ones, whereas the antioxidant activity was shown to be mainly related to the phenolic content. Our results indicate that the lettuce yields observed in the tested growth substrates are still not sufficient to ensure the self-sustainability of future space settlements. Exclusive input of pure water and manure does not appear to meet the minimum soil fertility requirements that are necessary to guarantee optimal crop development. However, it must be considered that pedogenesis is regulated by long processes that cannot be accomplished in a single lettuce cycle since the formation of fertile soil from disintegrated parental rock requires several chemical and physical alterations and continuous inputs of organic matter. Similarly, in the future extraterrestrial outpost, it will be feasible to gradually improve the regolith fertility with repeated cropping cycles and continuous inputs of organic substances composed partly by crew excrements and partly by the residues of previous crops. In addition, a start-up phase involving minimal nutrient supplementation and inoculation of plant growth-promoting rhizobacteria (PGPR) should be also investigated in future studies. In this regard, we can consider our results promising, as they demonstrated that by adding 30% of manure to pure regolith, it is possible to complete a lettuce cycle by feeding the plants with only pure water. However, further studies are needed with more lettuce genotypes to explore genotypic variation and make safe conclusions about the potential cultivation of the species in regolith.

## Data Availability Statement

The raw data supporting the conclusions of this article will be made available by the authors, without undue reservation.

## Author Contributions

YR contributed to conceptualization, visualization, supervision, and project administration. LD, AP, SP, AC, PA, GG, AR, and SD contributed to methodology, validation, formal analysis, investigation, and writing—original draft preparation. YR and SD contributed to funding acquisition and resources. LD and AP contributed to software and data curation. LD, AP, SP, AC, PA, GG, AR, SD, and YR contributed to writing, reviewing, and editing the manuscript. All authors contributed to the article and approved the submitted version.

## Conflict of Interest

The authors declare that the research was conducted in the absence of any commercial or financial relationships that could be construed as a potential conflict of interest.

## Publisher’s Note

All claims expressed in this article are solely those of the authors and do not necessarily represent those of their affiliated organizations, or those of the publisher, the editors and the reviewers. Any product that may be evaluated in this article, or claim that may be made by its manufacturer, is not guaranteed or endorsed by the publisher.
